# Snail and serpinA1 promote tumor progression and predict prognosis in colorectal cancer

**DOI:** 10.18632/oncotarget.3964

**Published:** 2015-04-29

**Authors:** Chae Hwa Kwon, Hye Ji Park, Jin Hwa Choi, Ja Rang Lee, Hye Kyung Kim, Hong-jae Jo, Hyun Sung Kim, Nahmgun Oh, Geun Am Song, Do Youn Park

**Affiliations:** ^1^ Department of Pathology, Pusan National University Hospital and Pusan National University School of Medicine, and BioMedical Research Institute, Pusan National University Hospital, Seo-Gu, Busan, Korea; ^2^ Department of Surgery, Pusan National University Hospital and Pusan National University School of Medicine, and BioMedical Research Institute, Pusan National University Hospital, Seo-Gu, Busan, Korea; ^3^ Department of Internal Medicine, Pusan National University Hospital and Pusan National University School of Medicine, and BioMedical Research Institute, Pusan National University Hospital, Seo-Gu, Busan, Korea

**Keywords:** colorectal cancer, snail, serpinA1, prognosis, fibronectin

## Abstract

The role of Snail and serpin peptidase inhibitor clade A member 1 (serpinA1) in tumorigenesis has been previously identified. However, the exact role and mechanism of these proteins in progression of colorectal cancer (CRC) are controversial. In this study, we investigated the role of Snail and serpinA1 in colorectal cancer (CRC) and examined the mechanisms through which these proteins mediate CRC progression. Immunohistochemical analysis of 528 samples from patients with CRC showed that elevated expression of Snail or serpinA1 was correlated with advanced stage, lymph node metastasis, and poor prognosis. Moreover, we detected a correlation between Snail and serpinA1 expression. Functional studies performed using the CRC cell lines DLD-1 and SW-480 showed that overexpression of Snail or serpinA1 significantly increased CRC cell invasion and migration. Conversely, knockdown of Snail or serpinA1 expression suppressed CRC cell invasion and migration. ChIP analysis revealed that Snail regulated serpinA1 by binding to its promoter. In addition, fibronectin mediated Snail and serpinA1 signaling was involved in CRC cell invasion and migration. Taken together, our data showed that Snail and serpinA1 promoted CRC progression through fibronectin. These findings suggested that Snail and serpinA1 were novel prognostic biomarkers and candidate therapeutic targets in CRC.

## INTRODUCTION

Snail is a family of zinc-finger transcription factors implicated in induction of the epithelial-mesenchymal transition (EMT) via suppression of E-cadherin expression, which disrupts normal epithelial cell-cell adhesion and facilitates invasion [1-3]. Enhanced expression of Snail has been found in a variety of cancer types, including breast, ovarian, prostate, lung, and gastric cancers, as well as melanoma, and has been reported to be frequently associated with invasiveness, metastasis, and poor prognosis [4-10].

In colorectal cancer (CRC), Snail expression is significantly elevated [11, 12] and has been reported to promote cancer progression by suppressing 15-PGDH expression [13]. Snail is also associated with downregulation of the vitamin D receptor and E-cadherin in CRC [14]. However, the tumorigenic effects and underlying mechanisms through which Snail mediates CRC are still not completely understood. Moreover, the results of clinical investigations are controversial. Roy et al. [11] and Pena et al. [14] have reported that the aberrant expression of Snail in tumors may be associated with metastatic ability, whereas Kroepil et al. [12] showed that there is no significant correlation between Snail expression in tumors and clinicopathological parameters or overall survival. Despite these findings, the clinical significance of Snail expression in CRC remains poorly understood.

Previously, we observed that Snail was correlated with prognosis in gastric cancer [15]. We have also reported that serpin peptidase inhibitor clade A member 1 (serpinA1) is a direct target of Snail in gastric cancer cells. SerpinA1, a type of serine protease inhibitor, has been reported to modulate invasive and metastatic capacity in lung cancer, gastric cancer, and CRC [16-18]. Moreover, serpinA1 mRNA expression is elevated in blood samples from patients with CRC patients and is an accurate biomarker for predicting prognoses in patients with CRC [19]. However, the mechanisms through which serpinA1 promote CRC progression are not yet known.

In this study, we identified Snail and serpinA1 as prognostic biomarkers of poor overall survival in CRC. In addition, we demonstrated that Snail and serpinA1 were important regulators of CRC cell invasion and migration through a pathway involving upregulation of fibronectin. Thus, our study provides important insights into the mechanisms of CRC pathogenesis.

## RESULTS

### Snail and serpinA1 were prognostic factors for patients with CRC

**Figure 1:**
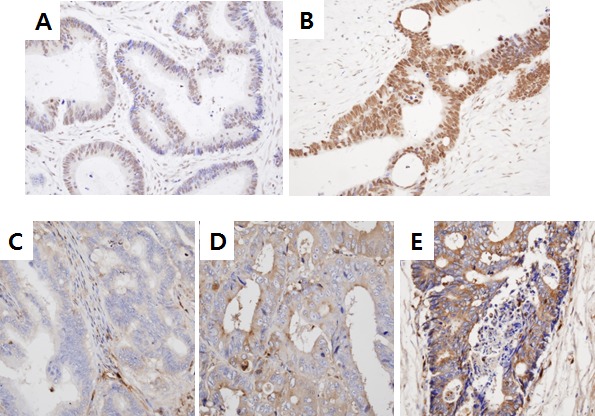
Snail and serpinA1 expression in patients with CRC Snail and serpinA1 expression levels were determined by IHC analysis in sections of TMAs. Weak (< 75%) **A.** or strong (≥ 75%) **B.** nuclear Snail immunostaining was detected in tumor cells. Negative **C.**, weak **D.**, or strong **E.** cytoplasmic staining for serpinA1 was observed in tumor cells.

To assess whether Snail and serpinA1 were involved in the progression of CRC, we first conducted immunohistochemical staining for Snail and serpinA1 in 528 and 522 CRC tissues, respectively, and analyzed the clinical relevance of Snail and serpinA1 expression. For Snail, only detectable nuclear staining was considered positive. Positive nuclear staining signals for Snail at levels of less than 75% and greater than or equal to 75% were observed in 73.7% (389/528) and 26.3% (139/528) of cases, respectively (Figure 1A and 1B). For serpinA1, cytoplasmic staining in tumor cells was considered positive. Negative (−) and low (+) or strong (++) cytoplasmic staining for serpinA1 were noted in 65.1% (340/522) and 34.9% (182/522) of cases, respectively (Figure 1C–1E). Furthermore, there was a significant positive correlation between the expression of Snail and serpinA1 (*P* < 0.0001, *r* = 0.217; data not shown).

**Figure 2:**
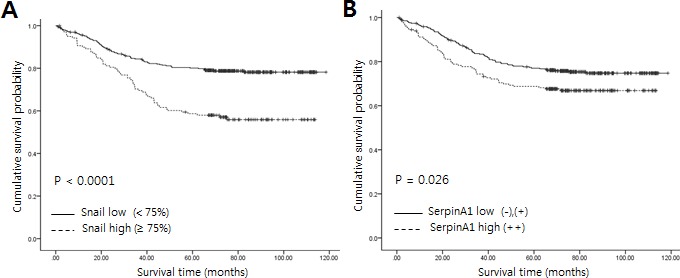
Relationship between expression of Snail or serpinA1 and clinical outcomes in patients with CRC Kaplan-Meier survival analysis was performed according to Snail or serpinA1 expression. **A.**, **B.** Linear relationships were observed between increased Snail or serpinA1 expression and shortened overall survival in CRC (*P* < 0.0001 and *P* = 0.023, respectively). *P*-values were calculated by the log-rank test.

Snail was overexpressed (≥ 75% positivity) in poorly differentiated CRC (*P* < 0.0001, Table 1), and the expression of Snail was also strongly elevated in patients with advanced stages (*P* = 0.009), perineural invasion (*P* = 0.002), lymphovascular emboli (*P* = 0.018), and lymph node metastasis (*P* = 0.001). Moreover, patients with at least 75% positive expression of Snail had significantly poorer survival rates than patients with less than 75% low expression (*P* < 0.0001, Figure 2A). On the basis of multivariate Cox regression analysis, the expression of Snail was an independent prognostic factor for overall survival (*P* < 0.0001, Table 2). Lymph node metastasis was also shown to be an independent prognostic factor for overall survival (*P* < 0.0001, Table 2).

**Table 1:** Relationship between Snail expression and clinicopathological characteristics in 528 patients with colorectal cancer

		Snail expression		*P* value
[No.]	< 75%	≥ 75%		
Age (years)	528	63.0 ± 0.56	63.5 ± 1.06	0.633
Size (cm)	528	5.12± 0.11	5.38 ± 0.19	0.246
Sex				
Male	308	229 (74.4)	79 (25.6)	0.676
Female	220	160 (72.7)	60 (27.3)	
Location				
Right Colon	129	91 (70.5)	38 (29.5)	0.353
Left Colon	399	298 (74.7)	101 (25.3)	
**Histological type**				**< 0.0001**
**Well**	**46**	**37 (80.4)**	**9 (19.6)**	
**Moderately**	**427**	**323 (75.6)**	**104 (24.4)**	
**Poorly**	**29**	**11 (37.9)**	**18 (62.1)**	
**Mucinous**	**26**	**18 (69.2)**	**8 (30.8)**	
**Invasion depth**				**0.009**
**T1**	**25**	**23 (92.0)**	**2 (8.0)**	
**T2**	**75**	**60 (80.0)**	**15 (20.0)**	
**T3**	**374**	**274 (73.3)**	**100 (26.7)**	
**T4**	**54**	**32 (59.3)**	**22 (40.7)**	
**Perineural invasion**				**0.002**
**Negative**	**330**	**258 (78.2)**	**72 (21.8)**	
**Positive**	**198**	**131 (66.2)**	**67 (33.8)**	
**Lymphovascular emboli**				**0.018**
**Negative**	**318**	**246 (77.4)**	**72 (22.6)**	
**Positive**	**210**	**143 (68.1)**	**67 (31.9)**	
**Lymph node metastasis**				**0.001**
**N0**	**282**	**220 (78.0)**	**62 (22.0)**	
**N1a(1)**	**72**	**55 (76.4)**	**17 (23.6)**	
**N1b(2–3)**	**84**	**61 (72.6)**	**23 (27.4)**	
**N2a(4–6)**	**49**	**34 (69.4)**	**15 (30.6)**	
**N2b(≥7)**	**41**	**19 (46.3)**	**22 (53.7)**	
Microsatellite status				0.486
MSS	466	340 (73.0)	126 (27.0)	
MSI-L	19	14 (73.7)	5 (26.3)	
MSI-H	43	35 (81.4)	8 (18.6)	

**Table 2:** Multivariate survival analysis with Cox regression model in 528 colorectal cancers

Variables	B	SE	HR (95% CI)	*P*
Age (<63 and ≥63)	−0.293	0.175	0.746 (0.530–1.050)	0.093
Lymph node metastasis (absent *vs*. present)	−1.114	0.172	0.328 (0.227–0.475)	<0.0001
Gender (male *vs*. female)	−0.001	0.170	0.999 (0.716–1.393)	0.993
Site (right *vs*. left colon)	0.241	0.188	1.272 (0.881–1.838)	0.199
Depth of invasion (T1,T2 *vs*. T3,T4)	−0.625	0.310	0.536 (0.292-0.983)	0.044
Snail (<75% and ≥75%)	−0.678	0.172	0.507(0.362–0.712)	<0.0001

Note: B, coefficient; HR, hazard ratio; CI, confidence interval.

SerpinA1 overexpression was correlated with mean tumor size (*P* = 0.014, Table 3). SerpinA1 was also frequently overexpressed in patients with advanced stages (*P* = 0.014) and lymph node metastasis (*P* = 0.006). Moreover, Kaplan-Meier survival analysis showed a strong correlation between serpinA1 expression and shorter overall survival (*P* = 0.023, Figure 2B). Taken together, these results showed that Snail and serpinA1 were associated with advanced clinical stage, lymph node metastasis, and poor prognosis in patients with CRC.

**Table 3:** Relationship between SerpinA1 expression and clinicopathological characteristics in 522 patients with colorectal cancer

		Snail expression		*P* value
[No.]	(−)(+)	(++)		
Age (years)	522	62.8 ± 0.62	63.6 ± 0.84	0.434
**Size (cm)**	**522**	**5.02± 0.12**	**5.54 ± 0.17**	**0.014**
Sex				
Male	303	197 (65.0)	106 (35.0)	0.947
Female	219	143 (65.3)	76 (34.7)	
Location				
Right Colon	126	86 (68.3)	40 (31.7)	0.399
Left Colon	396	254 (64.1)	142 (35.9)	
Histological type				0.269
Well	45	30 (66.7)	15 (33.3)	
Moderately	422	275 (65.2)	147 (34.8)	
Poorly	29	15 (51.7)	14 (48.3)	
Mucinous	26	20 (76.9)	8 (23.1)	
Invasion depth*				**0.014**
**T1**	**24**	**18 (75.0)**	**8 (25.0)**	
**T2**	**75**	**57 (76.0)**	**18 (24.0)**	
**T3**	**369**	**234 (63.4)**	**135 (36.6)**	
**T4**	**54**	**31 (57.4)**	**23 (42.6)**	
Perineural invasion				0.206
Negative	326	219 (67.2)	107 (32.8)	
Positive	196	121 (61.7)	75 (38.3)	
Lymphovascular emboli				0.304
Negative	314	210 (66.9)	104 (33.1)	
Positive	208	130 (62.5)	78 (37.5)	
**Lymph node metastasis**				**0.006**
**N0**	**278**	**195 (70.1)**	**83 (29.9)**	
**N1a(1)**	**72**	**42 (58.3)**	**30 (41.7)**	
**N1b(2–3)**	**83**	**58 (69.9)**	**25 (30.1)**	
**N2a(4–6)**	**48**	**26 (54.2)**	**22 (45.8)**	
**N2b(≥7)**	**41**	**19 (46.3)**	**22 (53.7)**	
Microsatellite status				0.565
MSS	460	296 (64.3)	164 (35.7)	
MSI-L	19	14 (73.7)	5 (26.3)	
MSI-H	43	30 (69.8)	13 (30.2)	

* Note: T1, T2 vs T3, T4,

### Snail and serpinA1 induced CRC cell invasion and migration

**Figure 3:**
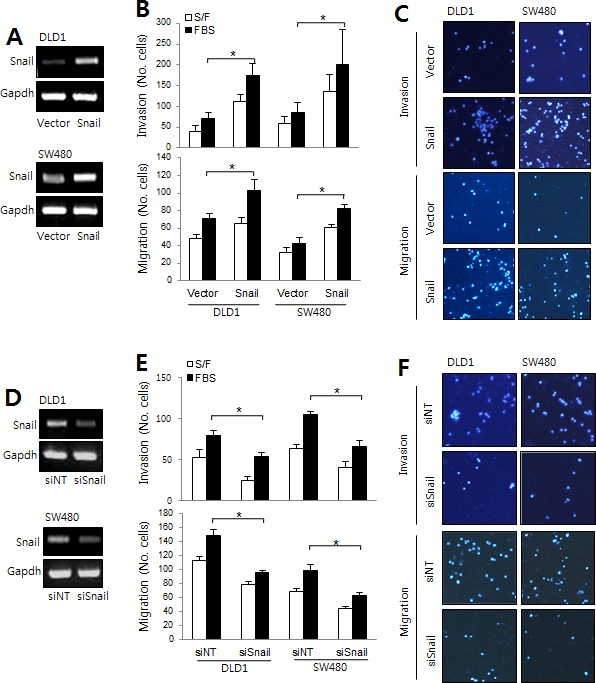
The roles of Snail in CRC **A.** DLD-1 and SW480 cells were transfected with empty vector or a Snail expression construct to analyze migration and invasion capacity. *Snail* mRNA levels were determined by RT-PCR. **B.** Graphs show the number of cells that invaded (top) and migrated (bottom) in the presence or absence of 1% FBS. *P < 0.05. **C.** Representative data are shown for cells that invaded (top) and migrated (bottom) in the presence of 1% FBS. **D.** DLD-1 and SW480 cells were transfected with nontargeting siRNA (siNT) or Snail siRNA (siSnail), and migration and invasion assays were performed. *Snail* mRNA levels were then determined by RT-PCR. **E.** Graphs show the number of cells that invaded (top) and migrated (bottom) in the presence or absence of 1% FBS. *P < 0.05. **F.** Representative data are shown for cells that invaded (top) and migrated (bottom) in the presence of 1% FBS.

Next, we tested whether the Snail/serpinA1 signaling pathway was involved in the progression of CRC. DLD-1 and SW-480 cells were transfected with a Snail expression construct or siRNA against Snail (siSnail) for evaluation of the effects of Snail on invasion and migration. The expression levels of Snail were assessed by real-time PCR (Figure 3A and 3D). Our results showed that overexpression of Snail significantly increased invasion and migration in DLD-1 and SW-480 cells (Figure 3B and 3C). In contrast, invasion and migration were decreased by Snail knockdown (Figure 3E and 3F).

**Figure 4:**
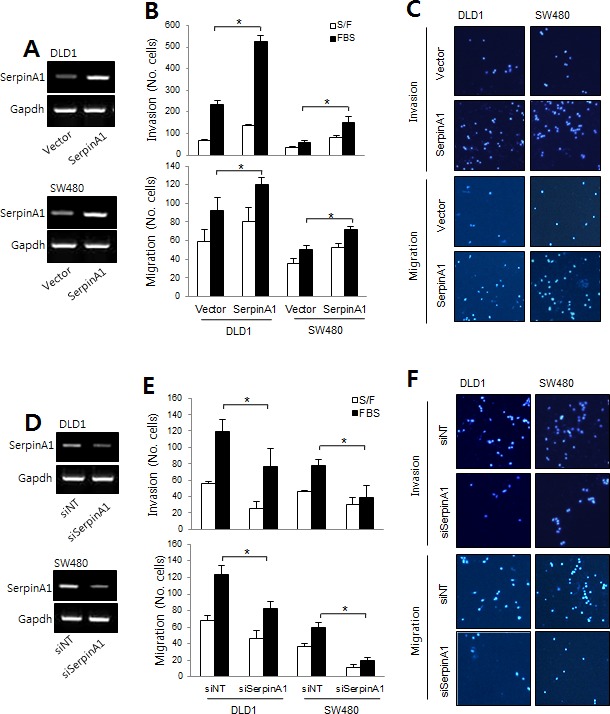
The roles of serpinA1 in CRC **A.** DLD-1 and SW480 cells were transfected with empty vector or a serpinA1 expression construct, and migration and invasion assays were performed. SerpinA1 mRNA levels were determined by RT-PCR. **B.** Graphs show the number of cells that invaded (top) and migrated (bottom) in the presence or absence of 1% FBS. *P < 0.05. **C.** Representative data are shown for cells that invaded (top) and migrated (bottom) in the presence of 1% FBS. **D.** DLD-1 and SW480 cells were transfected with nontargeting siRNA (siNT) or serpinA1 siRNA (siSerpinA1) for the migration and invasion assays. SerpinA1 mRNA levels were determined by RT-PCR. **E.** Graphs show the number of cells that invaded (top) and migrated (bottom) in the presence or absence of 1% FBS. *P < 0.05. **F.** Representative data are shown for cells that invaded (top) and migrated (bottom) in the presence of 1% FBS.

Similar experiments were performed to assess the role of serpinA1 in the progression of CRC. The expression levels of serpinA1 were analyzed by real-time PCR (Figure 4A and 4D). Our results showed that overexpression of serpinA1 also significantly increased the invasion and migration of DLD-1 and SW-480 cells (Figure 4B and 4C). Conversely, invasion and migration were decreased by serpinA1 knockdown (Figure 4E and 4F). These results suggested that Snail and serpinA1 were essential for conferring cancer-related phenotypes in DLD-1 and SW-480 cells.

### Snail regulated serpinA1 by binding to its promoter in CRC

**Figure 5:**
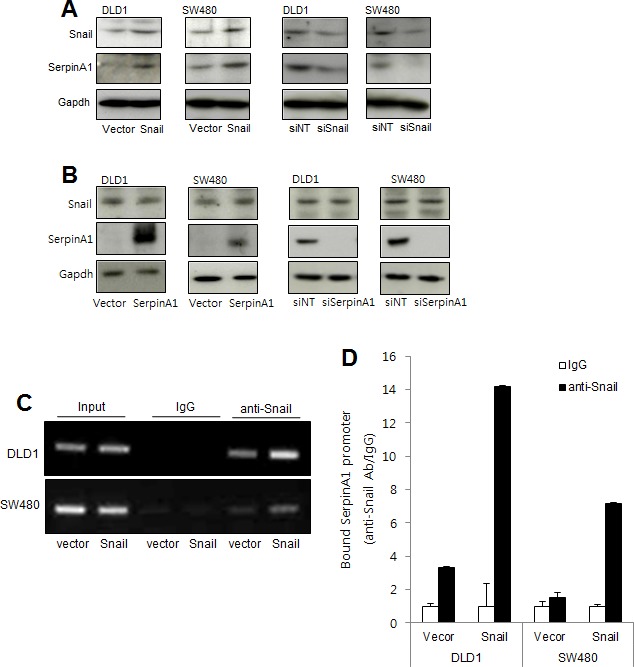
SerpinA1 was regulated by Snail **A.** DLD-1 and SW480 cells were transfected with pcDNA-Snail (Snail), control vector pcDNA (vector), Snail siRNA (siSnail), or nontargeting siRNA (siNT), and Snail and serpinA1 protein levels were evaluated by western blot analysis. **B.** DLD-1 and SW480 cells were transfected with pcDNA-serpinA1 (serpinA1), control vector pcDNA (vector), serpinA1 siRNA (siSerpinA1), or nontargeting siRNA (siNT), and western blot analysis was performed for detection of Snail and SerpinA1 expression. **C.** DLD-1 and SW480 cells were transfected with pcDNA-Snail (Snail) or control vector pcDNA (vector), and ChIP assays were performed. The presence of the serpinA1 promoter (−516/−4) was verified in immunoprecipitates with either mouse IgG or anti-Snail antibodies, and assay inputs were analyzed using real-time PCR. The samples were loaded on agarose gels. **D.** Data show promoter enrichment in the anti-Snail immunoprecipitate relative to IgG.

Since we had observed that Snail expression was correlated with serpinA1 expression in immunohistochemistry analysis, we investigate whether Snail could regulate serpinA1 expression. DLD-1 and SW-480 cells were transfected with Snail or serpinA1 expression constructs or siRNA targeting Snail or serpinA1. Overexpression of Snail increased serpinA1 expression, while knockdown of Snail decreased serpinA1 expression (Figure 5A). However, alteration of serpinA1 levels did not affect Snail expression (Figure 5B). These results suggested that serpinA1 may be regulated by Snail.

We previously reported that Snail regulates serpinA1 by binding to its promoter in gastric cancer [15]. Thus, to examine the molecular mechanisms through which Snail may regulate serpinA1 expression in CRC, we performed ChIP assays using Snail-overexpressing cells. In these cells, there was more than a four-fold increase in the precipitation of the serpinA1 promoter with anti-Snail antibodies than with IgG alone (Figure 5C and 5D). These results indicated that Snail regulated serpinA1 by binding to its promoter.

### Fibronectin mediated Snail and serpinA1 signaling in CRC

**Figure 6:**
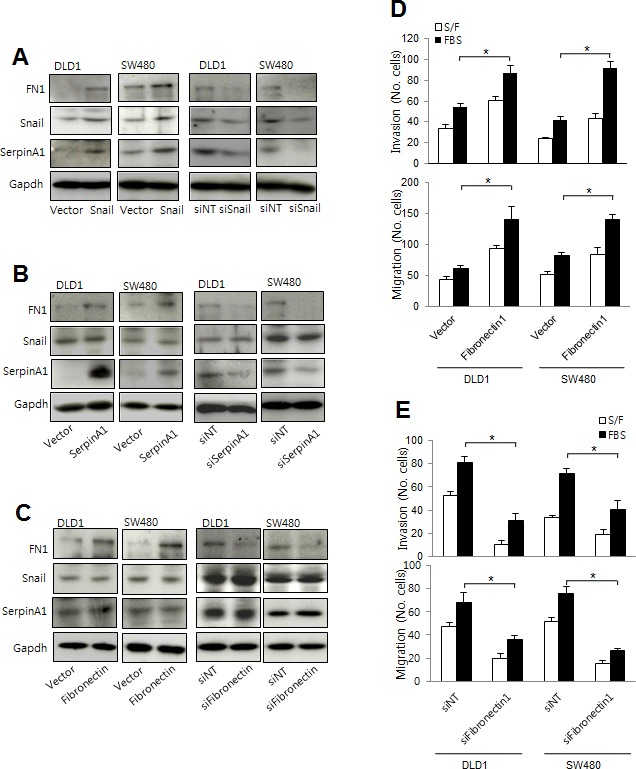
Snail and serpinA1 promoted tumor progression through fibronectin **A.** DLD-1 and SW480 cells were transfected with pcDNA-Snail (Snail), control vector pcDNA (vector), Snail siRNA (siSnail), or nontargeting siRNA (siNT), and Snail, serpinA1, and fibronectin protein levels were evaluated by western blot analysis. **B.** DLD-1 and SW480 cells were transfected with pcDNA-serpinA1 (SerpinA1), control vector pcDNA (vector), serpinA1 siRNA (siSerpinA1), or nontargeting siRNA (siNT), and Snail, serpinA1, and fibronectin protein levels were evaluated by western blot analysis. **C.** DLD-1 and SW480 cells were transfected with pcDNA-fibronectin (Fibronectin), control vector pcDNA (vector), fibronectin siRNA (siFibronectin), or nontargeting siRNA (siNT), and Snail, serpinA1, and fibronectin protein levels were evaluated by western blot analysis. **D.**, **E.** Invasion and migration assays were performed using transfected cells. Representative data are shown for cells that invaded (top) and migrated (bottom) in the presence of 1% FBS. *P < 0.05.

SerpinA1 has been reported to regulate the aggregation of fibronectin on surfaces of tumor cells, which may increase the probability of metastasis [21]. Thus, to further understand the mechanisms through which serpinA1 regulates CRC progression, we examined the effects of Snail and serpinA1 expression on fibronectin levels. Western blot analysis showed that the expression of fibronectin in DLD-1 and SW-480 cells was increased after transfection with Snail or serpinA1 expression constructs (Figure 6A). Conversely, knockdown of Snail or serpinA1 by transfection with the appropriate siRNA decreased the expression of fibronectin (Figure 6B). Importantly, alteration of fibronectin expression did not change levels of Snail or serpinA1 (Figure 6C).

Next, we checked whether fibronectin was also involved in the progression of CRC. Invasion and migration were increased in cells transfected with the fibronectin expression construct (Figure 6D). Conversely, knockdown of fibronectin expression using specific siRNA attenuated cell invasion and migration (Figure 6E). These results suggested that fibronectin functioned downstream of Snail and serpinA1 signaling pathways and was necessary for CRC progression.

### The roles of Snail and serpinA1 in other types of human cancer cells

**Figure 7:**
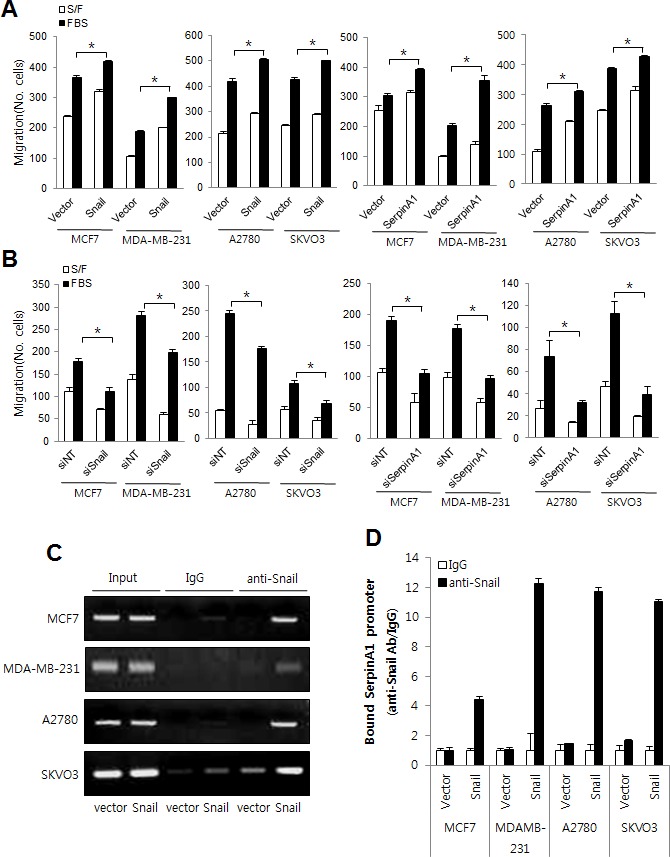
The roles of Snail and serpinA1 in other human cancer cell lines **A.**, **B.** MCF7, MDA-MB-231, A2780, and SKVO3 cells were transfected with pcDNA-Snail, pcDNA-serpinA1, Snail siRNA, or serpinA1 siRNA, and migration assays were performed using these cells. Graphs show the number of cells that migrated in the presence or absence of 1% FBS. *P < 0.05. **C.** MCF7, MDA-MB-231, A2780, and SKVO3 cells cells were transfected with pcDNA-Snail (Snail) or control vector pcDNA (vector), and ChIP assays were performed. The presence of the serpinA1 promoter (−516/−4) was verified in immunoprecipitates with either mouse IgG or anti-Snail antibodies, and assay inputs were analyzed using real-time PCR. The samples were loaded on agarose gels. **D.** Data shows promoter enrichment in the anti-Snail immunoprecipitate relative to IgG.

Since Snail and serpinA1 are expressed not only in CRC but also in other types of cancer, [3, 16-18] we also examined the role of Snail and serpinA1 in breast and ovarian cancer cells (MCF7, MDA-MB-231, A2780, and SKVO3 cells) by modulating their expression. The results showed that Snail and serpinA1 induced migration in both breast and ovarian cancer cells (Figure 7A and 7B and Supplementary Figures 1 and 2). In addition, the results of ChIP assays showed that Snail may also modulate serpinA1 expression by binding to the serpinA1 promoter in breast and ovarian cancer cells (Figure 7C and 7D and Supplementary Figures 3). These results indicated that serpinA1 may be regulated by Snail and that Snail and serpinA1 signaling induces tumor progression in a variety of cancer cells.

## DISCUSSION

CRC is a major cause of morbidity and mortality worldwide. The incidence of CRC in Korea has increased dramatically over the past few decades, while the incidence rates of more common cancers, such as stomach and liver cancers, have decreased [22]. However, the biological mechanisms driving poor clinical outcomes remain incompletely understood. Therefore, it is necessary to develop prognostic biomarkers and novel therapeutic targets for the treatment and prevention of CRC. In this study, we evaluated Snail and serpinA1 as potential biomarkers in CRC.

Snail is a zinc-finger transcription factor that is known to induce the EMT [23] and was originally shown to be expressed in invasive carcinoma cells [2, 3]. However, some groups have found no evidence of any association between Snail expression in tumors and clinicopathological parameters or overall survival in CRC in relatively small cohorts [12, 24]. Thus, in this study, we analyzed the clinical significance of Snail expression in CRC development and progression using immunohistochemical analysis in a larger series. Our results showed that Snail was correlated with clinical stage, perineural invasion, lymphovascular emboli, lymph node metastasis, and overall survival in CRC patients. Importantly, further multivariate analyses of our TMA showed that the level of Snail expression was the only significant factor correlated with poor prognosis. Further investigation revealed that overexpression of Snail increased CRC cell invasion and migration, whereas knockdown of Snail attenuated CRC cell invasion and migration. These results indicated that Snail mediated the invasive and migratory capacity of CRC cells, affecting the metastatic potential of the cells. Interestingly, Snail also induced migration in the other cancer cell lines tested in the present study. Our results are in concordance with other studies, which showed that Snail-expressing cells acquire migratory and invasive properties [25, 26, 27].

Snail was reported to enhance invasiveness and metastasis through direct repression of E-cadherin transcription [25] or upregulation of metalloproteinases, such as MMP-2 and MMP-9, that are involved indirectly in the degradation of the basement membrane and extracellular matrix [26, 27]. In a previous study, we found that Snail regulates the expression of serpinA1 and is involved in gastric cancer progression [15, 20]. In this study, immunohistochemical analysis revealed that Snail overexpression was correlated with elevated levels of serpinA1 in CRC. Moreover, serpinA1 was upregulated in CRC cells overexpressing Snail and downregulated in CRC cells exhibiting Snail knockdown. Further analysis using ChIP assays revealed that Snail regulated the expression of serpinA1 through directly binding to the serpinA1 promoter not only in CRC cells but also in breast and ovarian cancer cells. These results show a significant relationship between serpinA1 and Snail in cancer cells and improve our understanding of the mechanism through which Snail is involved in tumor progression.

SerpinA1 is a protease inhibitor that functions as a serum trypsin inhibitor [28]. The serum levels of serpinA1 are higher in cancer patients than in healthy patients [29-31]. In addition, serpinA1 is related to the distant metastasis of various cancers, including ovarian, cervical breast, and lung cancers [32-34]. Consistent with this, we found that serpinA1 was significantly correlated with stage and lymph node metastasis in CRC. In addition, increase serpinA1 expression was related to poor prognosis in patients with CRC patients, and serpinA1 signaling regulated CRC cell motility and invasiveness. Further supporting this, serpinA1 has been reported to be associated with metastasis in CRC [35]. Additionally, a previous study showed that the serum levels of serpinA1 are significantly higher in patients with CRC than in healthy subjects [19]. Moreover, we also observed that serpinA1 promoted migration in breast and ovarian cancer cell lines. These results suggested that serpinA1 plays an important role in tumor progression, and our current data provided evidence of the key role of serpinA1 as a regulator of invasion and migration in CRC cells for the first time.

Although the mechanism of serpinA1 in tumor progression is not fully elucidated, it has been shown to promote lung colonization via fibronectin assembly [21]. In this study, we showed that fibronectin expression was regulated by Snail and serpinA1, suggesting that fibronectin functioned downstream of Snail and serpinA1. SerpinA1 may prevent the disruption of cell surface-fibronectin connections by inhibiting chymase activity, thereby mediating the assembly of fibronectin [36, 37]. Snail is also required for fibronectin activation in epithelial cells undergoing the EMT [38]. Thus, we cannot exclude the possibility that Snail regulates fibronectin expression directly. Further studies are required to elucidate the precise mechanisms through which Snail and serpinA1 mediate fibronectin expression.

Functionally, upregulation of fibronectin in CRC cells promoted invasion and migration, while suppression of fibronectin expression blocked cell invasion and migration, highlighting the role of fibronectin in CRC cell invasion and migration. These results are consistent with the many previous works showing that fibronectin contributes to tumor progression in breast, lung, and thyroid cancer through the activation of multiple oncogenic pathways, such as Akt, extracellular signal-regulated kinase, and signal transducer and activator of transcription 3 [39-41]. In our study, we identified a novel mechanism through which fibronectin mediated the motility of CRC cells via Snail and serpinA1. Thus, our data provide important insights into the molecular mechanisms and signaling events regulating CRC cell motility and progression.

In summary, we found that Snail and serpinA1 expression were associated with advanced stage, lymph node involvement, and poor prognosis in patients with CRC. We also provided evidence that Snail and serpinA1 induced CRC cell invasion and migration by upregulation of fibronectin, as well as migration of other cancer cells, demonstrating a novel signaling mechanism involved in tumor progression. These results suggested that Snail and serpinA1 may be useful biomarkers in the clinical setting and new therapeutic targets for development of novel therapeutic modalities in cancer management.

## MATERIALS AND METHODS

### Cell lines and transfection

The human cancer cell lines were obtained from the Korean Cell Line Bank (Seoul, South Korea) or ATCC (VA, USA). The human colon cancer cell lines DLD-1 and SW-480 were cultured in RPMI1640 medium. The human breast cancer cell lines MCF7 and MDA-MB-231 and the human ovarian cancer cell lines A2780 and SKVO3 were maintained in DMEM. All media were supplemented with 10% fetal bovine serum (FBS; Gibco; Thermo Scientific Inc.; PA, USA), 100 U/mL penicillin, and 100 μg/mL streptomycin (Sigma-Aldrich; MO, USA). All cells were maintained at 37°C in an atmosphere containing 5% CO_2_.

For overexpression of genes, cells were grown to 60–70% confluence and transfected with the pcDNA Snail-Myc, pcDNA SerpinA1-Myc vectors or pcDNA fibronectin-Myc vectors (Origene; MD, USA), or with the pcDNA-3.1 vector as a control, with Lipofectamine 2000 (Invitrogen; Life Technologies; NY, USA) according to the manufacturer's protocol. Cells were maintained in complete medium for 24 hours before the assays were performed.

For knockdown of genes, cells were transfected with Snail or serpinA1 smartpool short interfering RNA (siRNA) or with non-targeting siRNA as a control (Dharmacon; Thermo Scientific Inc.; PA, USA), using Lipofectamine RNAiMAX reagent (Invitrogen) according to the manufacturer's instructions. The siRNA sequences were as follows: Snail siRNA, 5′-GCGAGCUGCAGGACUCUAA-3′, 5′-AAUCGGAAGCCUAACUACA-3′, 5′-GUGACUAACUACUGCAAUAA-3′, 5′-GAGUAAUGGCUGUCACUUG-3′; serpinA1 siRNA, 5′-GAACUCACCCACGAUAUCA-3′, 5′-GAUGAAGCGUUUAGGCAUG-3′, 5′-CCUAUGAUCUGAAGAGCGU-3′, 5′-CCAAGAAACAGAUCAACGA-3′; fibronectin siRNA, 5′-GAACUCACCCACGAUAUCA-3′, 5′-GAUGAAGCGUUUAGGCAUG-3′, 5′-CCUAUGAUCUGAAGAGCGU-3′, 5′-CCAAGAAACAGAUCAACGA-3′;.

### Real time reverse transcription (RT)-PCR analysis

Total RNA was extracted from gastric cancer cells, using the TRIzol reagent (Invitrogen), following the manufacturer's instructions. RNA was reverse transcribed with SuperScript II (Invitrogen) and cDNA was amplified with each primer and visualized with SYBR Green (Applied Biosystems; Life Technologies; NY, USA), using the fluorescence reader Corbett Rotor-Gene 6000 (Qiagen Inc.; CA, USA). The primers used are the following:: glyceraldehyde 3-phosphate dehydrogenase (GAPDH), 5′-TCCATGACAACTTTGGTATCG-3′, 5′-TGTAGCCAAATTCGTTGTCA-3′; Snail, 5′-TCTTCCTCTCCATACCTG-3′, 5′-CATAGTTAGTCACACCTCGT-3′; and serpinA1, 5′-GGACACCGAGGAAGAGGA-3′, 5′-TCAGGCAGGAAGAAGATGG-3′. The following thermal cycler program was used: denaturation for 30 s at 95°C; annealing for 30 s at 52°C, depending on the primers used; and extension for 30 s at 72°C. The number of PCR cycles was determined for each gene and ranged from 25 to 35. Data were normalized to GAPDH, and mRNA abundance was calculated using the 2^−ΔΔCT^ method. The PCR products were confirmed by mobility on gel electrophoresis.

### Chromatin immunoprecipitation (ChIP) assays

ChIP assays were performed using the EZ-ChIP kit (Merck Millipore; Darmstadt, Germany). Cells were cross-linked with 2% formaldehyde for 10 min at 37°C and washed in ice-cold PBS. Unreacted formaldehyde was quenched with 200 mM glycine, and then cells were washed with PBS and resuspended in SDS lysis buffer containing protease inhibitors. Samples were sheared by sonication on ice, diluted in dilution buffer with inhibitors, and precleared with agarose G beads. A portion of the supernatant was stored as input, and the remaining supernatant was divided for immunoprecipitation and incubated with anti-Snail (R&D system; MN, USA) or IgG as a negative control overnight at 4°C with agitation. Immune complexes were captured using ProteinA-Sepharose, then washed sequentially in low-salt buffer, high-salt buffer, twice in LiCl buffer, then twice in TE buffer. Protein was eluted from the beads in fresh elution buffer, cross-linking was reversed overnight at 65°C in the presence of NaCl, and then samples were ethanol-precipitated. Following centrifugation, pellets were resuspended in TE buffer and incubated sequentially with 50 μg/mL RNase A (30 min) and 100 μg/mL proteinase K (1 h). DNA was purified by washing with elution buffer and centrifugation and then finally analyzed by real-time PCR. The primer sequence was 5′-AAAGAGCAGGACCCCAAAT-3′ and 5′-TCCACCCGAAGTCTACTTCC-3′.

### Cell migration and invasion assays

Gastric cancer cells were harvested with 0.05% trypsin containing 0.02% EDTA (Sigma-Aldrich) and suspended in RPMI medium. For the migration assay, membrane filters (8-μm pore size) in disposable 96-well chemotaxis chambers (Neuro Probe; Gaithersburg, MD, USA) were pre-coated with 5 mg/mL fibronectin for 4 h at room temperature. Cells (3 × 10^3^ cells/well) were loaded into the upper chambers, and 1% FBS was loaded into the lower chamber. After 24 h of incubation, non-migrating cells were removed from the upper chamber with a cotton swab, and the cells on the lower surface of the insert were stained with Hoechst33342 (Sigma-Aldrich). Migrated cells were counted under a fluorescence microscope at 10× magnification.

For the invasion assay, 3 × 10^4^ cells/well were seeded in the upper chamber, which was coated with 30 μL of Matrigel (1 mg/mL in cold medium; BD Transduction Laboratories; NJ, USA). Serum-free medium containing 1% FBS or control vehicle was added to the lower chamber. After 24 h of incubation, non-invading cells were removed from the upper chamber with a cotton swab, and cells on the lower surface of the insert were stained with Hoechst33342 (Sigma-Aldrich). Invasive cells were counted under a fluorescence microscope at 10× magnification.

### Western blot analysis

Cells were harvested and disrupted in lysis buffer (1% Triton X-100, 1 mM EGTA, 1 mM EDTA, 10 mM Tris-HCl at pH 7.4, and protease inhibitors). Cell debris was removed via centrifugation at 10,000 × *g* for 10 min at 4°C. The resulting supernatants were resolved using SDS-PAGE and transferred onto nitrocellulose membranes. The membranes were blocked with 5% non-fat dried milk at room temperature for 30 min and incubated with anti-Sanil (AF3639; R&D system), anti-serpinA1 (HPA000927; Sigma-Aldrich), anti-fibronectin (HPA027066; Sigma-Aldrich) and anti-GAPDH. The membranes were then washed and incubated with horseradish peroxidase-conjugated secondary antibody. Signals were visualized using enhanced chemiluminescence (Amersham; Buckinghamshire, UK).

### Immunohistochemistry and analysis of clinicopathological and prognostic significance

We studied a cohort of 528 CRC patients who received resection of the primary tumor at the Pusan National University Hospital (PNUH) between 2003 and 2008. Standard formalin-fixed and paraffin-embedded sections were obtained from the Department of Pathology and the National Biobank of Korea, PNUH. All samples from the National Biobank of Korea were obtained with informed consent under institutional review board-approved protocols.

Methods of immunohistochemistry have previously been described for Snail [20] and SerpinA1 [15]. Snail staining was considered positive when nuclear staining was detectable and graded as < 75 % and ≥ 75 % positivity, which has been described in our previous study [20]. SerpinA1 staining in tumor cells was considered positive. SerpinA1 immunostaining was graded as follows: negative (−) for no stainig, (+) for any staining, (++) for strong staining.

Clinicopathological features were analyzed for differences in Snail or serpinA1 expression using the Student's *t*-test, the χ^2^ test, or Fisher's exact test. The relationships between expression of Snail and serpinA1 were assessed with a Spearman rank correlation coefficient. Cumulative survival plots were obtained using the Kaplan-Meier method, and significance was compared using the log-rank test. Statistical significance was set at *P* < 0.05. Multivariate analyses were carried out using Cox proportional hazards regression. Statistical calculations were performed using SSPS version 10.0 for Windows (SPSS Inc.; Chicago, IL, USA).

## SUPPLEMENTARY FIGURES AND TABLES


