# Supplementary material for: Snail and serpinA1 promote tumor progression and predict prognosis in colorectal cancer
Source: Oncotarget. 2015 Apr 29;6(24):20312–26. doi: 10.18632/oncotarget.3964 (PMC4653007; doi:10.18632/oncotarget.3964)
Supplement: Supplementary file 1 [file oncotarget-06-20312-s001.pdf]

Snail and serpinA1 promote tumor progression and predict prognosis in colorectal cancer

Supplementary Material

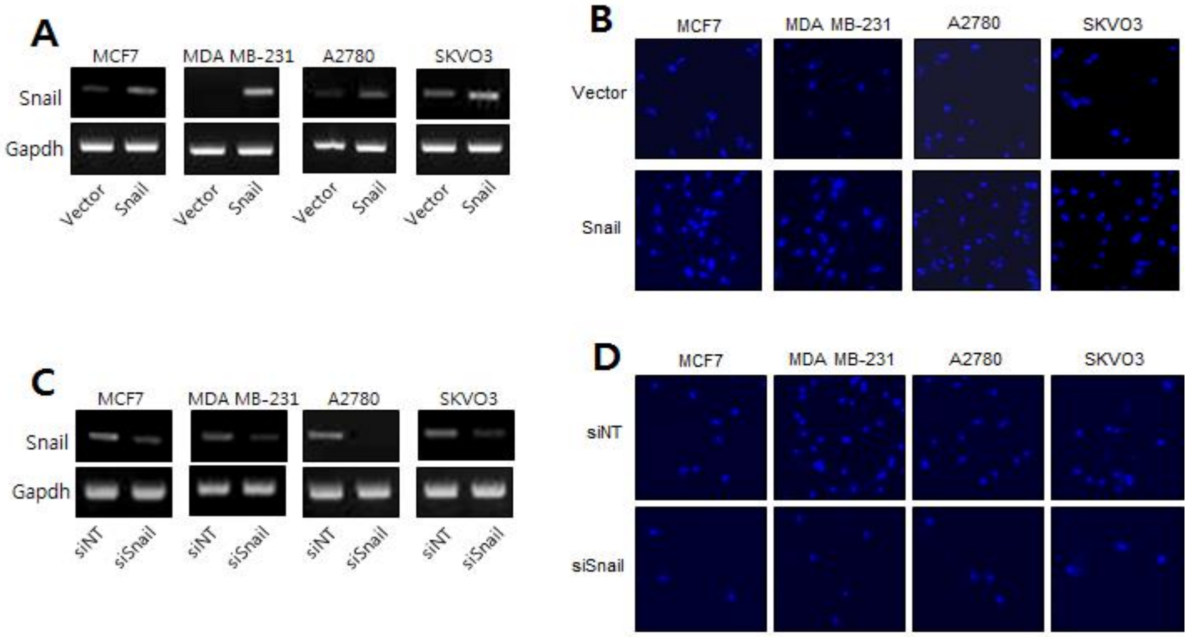

Supplementary figure 1. Snail is involved in the progression of breast and ovarian cancer cells. (**A**) MCF7, MDA-MB-231, A2780, and SKVO3 cells were transfected with empty vector or a Snail expression construct, and migration assays were performed. Snail mRNA levels were determined by RT-PCR. (**B**) Representative data are shown for cells that migrated in the presence of 1% FBS. (**C**) MCF7, MDA-MB-231, A2780, and SKVO3 cells were transfected with nontargeting siRNA (siNT) or Snail siRNA (siSnail) for the migration assays. Snail mRNA levels were determined by RT-PCR. (**D**) Representative data are shown for cells that migrated in the presence of 1% FBS.

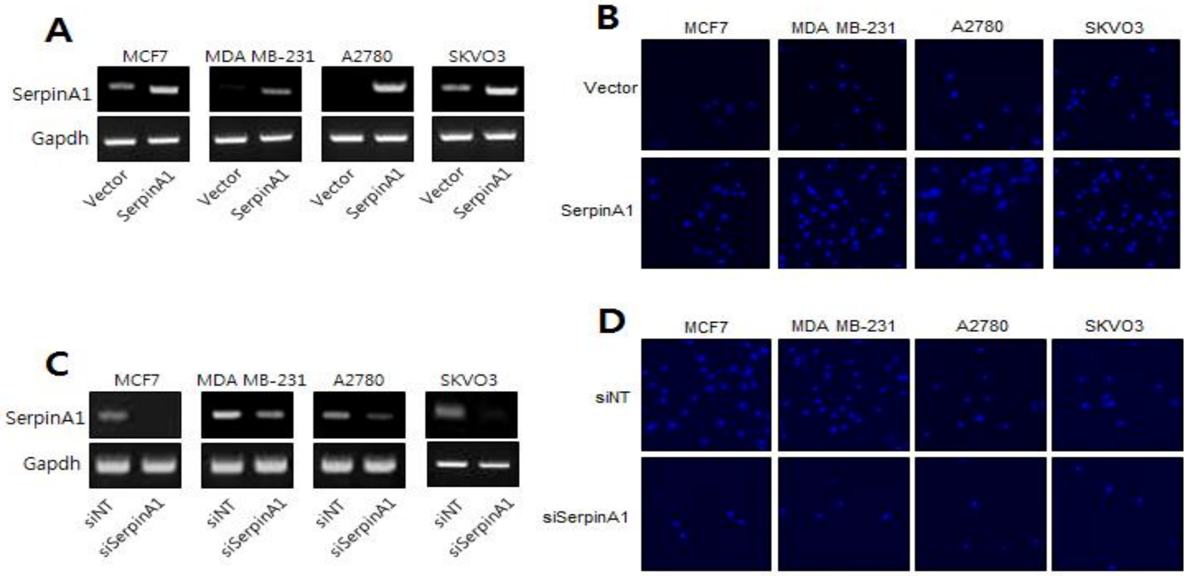

Supplementary figure 2. SerpinA1 is involved in the progression of breast and ovarian cancer cells. (**A**) MCF7, MDA-MB-231, A2780, and SKVO3 cells were transfected with empty vector or a serpinA1 expression construct, and migration assays were performed. SerpinA1 mRNA levels were determined by RT-PCR. (**B**) Representative data are shown for cells that migrated in the presence of 1% FBS. (**C**) MCF7, MDA-MB-231, A2780, and SKVO3 cells were transfected with nontargeting siRNA (siNT) or serpinA1 siRNA (siSerpinA1) for the migration assays. SerpinA1 mRNA levels were determined by RT-PCR. (**D**) Representative data are shown for cells that migrated in the presence of 1% FBS.

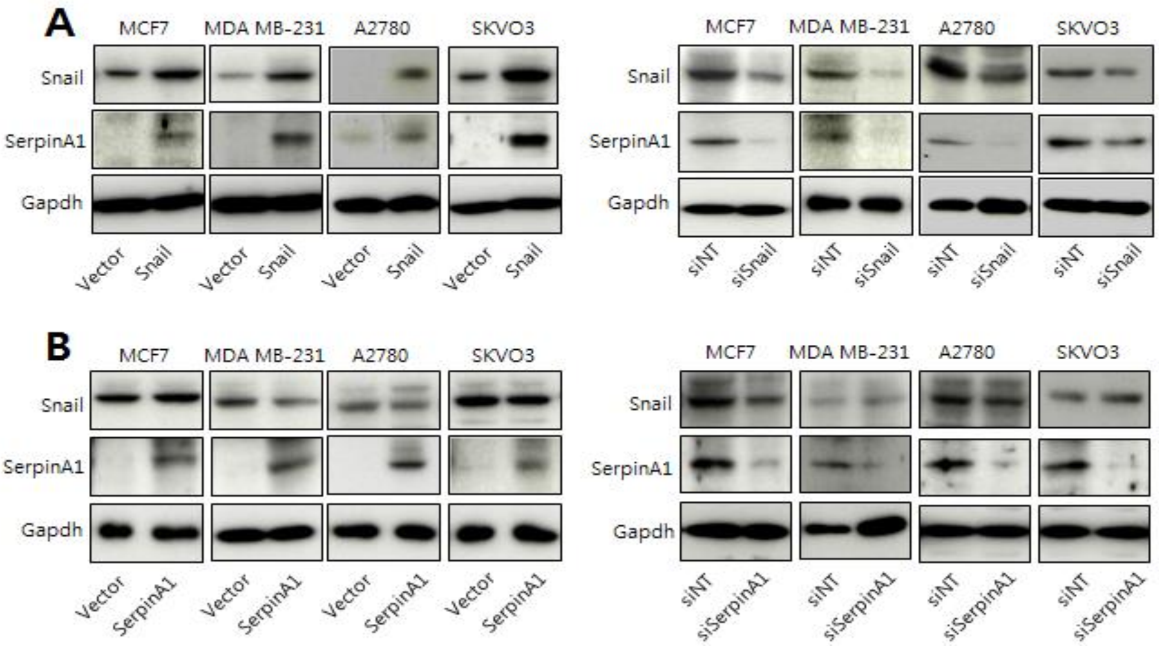

Supplementary figure 3. Expression of serpinA1 was regulated by Snail in breast and ovarian cancer cells. (**A**) MCF7, MDA-MB-231, A2780, and SKVO3 cells were transfected with pcDNA-Snail (Snail), control vector pcDNA (vector), Snail siRNA (siSnail), or nontargeting siRNA (siNT), and Snail and serpinA1 protein levels were evaluated by western blot analysis. (**B**) MCF7, MDA-MB-231, A2780, and SKVO3 cells were transfected with pcDNA-serpinA1 (serpinA1), control vector pcDNA (vector), serpinA1 siRNA (siSerpinA1), or nontargeting siRNA (siNT), and western blot analysis was performed for detection of Snail and SerpinA1 expression.
